# The Signaling Networks of TIM-3, TGF-β, and STING in Glioblastoma

**DOI:** 10.3390/cells15110991

**Published:** 2026-05-28

**Authors:** Farah Ahmady-Nield, Blaine M. H. Carnie, Grace E. C. Anderson, Emerson Achari, Amit Sharma, Adrian A. Achuthan, George Kannourakis, Rodney B. Luwor

**Affiliations:** 1 Fiona Elsey Cancer Research Institute, Ballarat, VIC 3350, Australia; farah@fecri.org.au (F.A.-N.); blaine.carnie@student.unimelb.edu.au (B.M.H.C.); grace.anderson.1@student.unimelb.edu.au (G.E.C.A.); george@fecri.org.au (G.K.); 2 Federation University Australia, Ballarat, VIC 3350, Australia; 3 Department of Surgery, The University of Melbourne, The Royal Melbourne Hospital, Parkville, VIC 3050, Australia; 4 Department of Medicine, The University of Melbourne, The Royal Melbourne Hospital, Parkville, VIC 3050, Australia; e.achari@student.unimelb.edu.au (E.A.); aaa@unimelb.edu.au (A.A.A.); 5 Department of Integrated Oncology, Center for Integrated Oncology (CIO) Bonn, University Hospital Bonn, 53127 Bonn, Germany; amit.sharma@ukbonn.de; 6 Department of Neurosurgery, University Hospital Bonn, 53127 Bonn, Germany; 7 Huagene Institute, Kecheng Science and Technology Park, Pukou District, Nanjing 211806, China

**Keywords:** glioblastoma, TIM-3, TGF-β, STING, pathways, immunosuppression, T cell exhaustion

## Abstract

Glioblastoma is the most aggressive form of brain tumor resulting in low overall patient survival rates of 12–15 months post diagnosis. Several factors contribute to the complexity of the tumor, including tumor heterogeneity, blood–brain barrier complications, genetic defects, cancer stem cell generation, and immune evasion. These factors can result in the progression of glioblastoma and are controlled by signaling pathways. Some of the signaling pathways involved in glioblastoma progression include ERK, NF-κB, Wnt, and PI3K/AKT/mTOR. Our and others’ previous studies have found that TIM-3 and TGF-β signaling is altered in glioblastoma patients and may contribute to cancer progression. Immune promoting pathways such as STING have also been studied in glioblastoma to enhance anti-tumor immunity; however the interconnecting roles of these pathways are not well described. This review highlights the role of these three key cancer-related pathways in glioblastoma and their mechanistic link. Better understanding these links may result in improved treatment targets or disease progression biomarkers.

## 1. Introduction

Glioblastoma is the most common and aggressive form of cancer originating in the brain. It is classified as a grade IV isocitrate dehydrogenase (IDH)-wildtype astrocytoma and features characteristics such as microvascular proliferation, EGFR gene amplification, and TERT promoter mutation [1,2]. Median overall survival (OS) rates for patients are low, between 12 and 15 months from diagnosis. Despite this low survival rate, the treatment regimen also known as the Stupp Protocol has remained largely unchanged for almost two decades. This protocol consists of surgical resection succeeded by adjuvant radiotherapy (60 Gray (Gy)) and adjuvant temozolomide (TMZ) (75 mg/m^2^ pre- and post- initial radiotherapy), followed by daily irradiation at 2 Gy for 30 days [3,4]. However, relapse in glioblastoma patients inevitably results in a five-year OS rate of ~5% [5,6]. Some factors which contribute to patient relapse include tumor heterogeneity, the blood–brain barrier (BBB) making it difficult to administer therapies, genetic defects, the development of glioblastoma stem cells, and immune evasion [7,8,9,10]. Signaling pathways play a pivotal role in these processes, hence a better understanding of these pathways in the context of glioblastoma will allow for ways to combat the disease.

Several factors can result in glioblastoma progression, including changes in molecular mechanisms and pathways. Pouyan et al. have comprehensively reviewed several key pathways associated with glioblastoma and their implementation as current and possible treatment strategies [11]. These pathways include epidermal growth factor receptor (EGFR), nuclear factor kappa-light-chain enhancer of activated B cells (NF-κB), Wnt, Phosphoinositide 3-kinase/Protein kinase B/Mammalian target of rapamycin (PI3K/AKT/mTOR), P14ARF/MDM2/p53, ATM/Chk2/p53, retinoblastoma (RB), sonic hedgehog, mitogen-activated protein kinase (MAPK), transforming growth factor-beta (TGF-β), T-cell immunoglobulin and mucin-domain containing protein-3 (TIM-3), and stimulator of interferon genes (STING) pathways, and genetic mutations [11,12,13]. Here we review three key cancer-related pathways, TGF-β, TIM-3, and STING pathways in glioblastoma and explore their intertwined crosstalk.

## 2. TIM-3

### 2.1. TIM-3 Background

T-cell immunoglobulin and mucin-domain containing protein-3 (TIM-3), also known as Hepatitis A virus receptor 2 (HAVCR2) is an immune checkpoint molecule that is part of the TIM gene family (TIM-1, TIM-3, TIM-4) [14]. TIM-3 was initially found on interferon-γ (IFN-γ) producing Th1 and Th17 (CD4+), and Tc1 (CD8+) T cells [15,16,17] in both regular [15,18] and tumor immune-cell environments in humans and mice [19,20]. It has since been identified on the surface of regulatory (Treg) cells and innate immune cells including natural killer (NK) cells, macrophages, and dendritic cells (DCs) [21,22], suggesting its role across multiple layers of immunity. In the context of cancer, TIM-3 can also be expressed on tumor cells, where it promotes tumor cell migration and invasion and pro-tumor macrophage polarization via PTEN and NF-κB/IL-6/signal transducer of transcription 3 (STAT3) signaling pathways in glioblastoma [23,24,25] and other cancers [26,27]. On immune cells, TIM-3 functions as a type 1 co-inhibitory transmembrane glycoprotein to negatively regulate the proliferation of immune cells such as CD8+ T cells, and their cytokine production following an immune response [28,29]. Other methods of immunosuppression by TIM-3 include its elevated expression on regulatory T (Treg) cells and thus its enhanced suppressiveness via greater IL-10 production [30]. Additionally, the expression of TIM-3 on macrophages is linked to M2 polarization [31,32].

The TIM-3 complex consists of an N-Terminal IgV domain, a mucin stalk/domain, a transmembrane, and a cytoplasmic tail [33]. The cytoplasmic tail of TIM-3 has two tyrosine (Tyr) components; Tyr256 and Tyr263 which are important in TIM-3 signal transduction. Upon T cell activation, TIM-3 complex is transported from lipid rafts, where they reside, to immune synapses [34]. There are four known ligands of the TIM-3 receptor, each of which can bind to different regions of the TIM-3 IgV domain [35,36,37]. Two are cell surface ligands (carcinoembryonic antigen-related cell adhesion molecule-1 (CEACAM-1) [38] and phosphatidylserine (PtdSer) [39]) and the other two are soluble ligands (galectin-9 [18] and high mobility group box 1 (HMGB1) [36]). TIM-3 has been associated with several molecules at the immunological synapse, which determines the function of the cell. An example of this is the colocalization of TIM-3 with receptor phosphatases CD45 and CD148 on T cells, which is further amplified when a galectin-9 ligand is bound to the TIM-3 receptor [34,40].

When the TIM-3 receptor is unbound, the adapter protein HLA-B associated transcript 3 (BAT3; or BAG6) binds to Tyr256 and Tyr263 in the cytoplasmic tail, and this process employs the src-kinase lymphocyte-specific protein tyrosine kinase (LCK) [34,41], and ultimately allows for the activation of the immune cell and their functional preservation. When a ligand binds to the TIM-3 receptor, the Tyr256 and Tyr263 components phosphorylate and remove BAT3 off the TIM-3 complex. This activates a series of downstream signaling cascades including the binding of the src-kinase Fyn to the cytoplasmic tail [42], resulting in the immune cells becoming functionally exhausted [43]. A proteomics study by Zhai et al. has shown that other intracellular molecules such as VAV1, LCK, GRB2, SHP1, CEBLB, PI3KR1, and UBASH3A also have the ability to bind to the cytoplasmic tail of TIM-3 complex, tested on CD4+ T cells [40]; however, binding studies for most of these individual proteins and their functional outcome on the cell remains unclear.

In the context of cancer, TIM-3 is generally upregulated, acts as a checkpoint molecule, and is linked to poor patient outcomes due to the upregulation of the TIM-3 ligands and the inevitable exhaustion of immune cells [44,45,46,47,48,49,50,51]. This is also seen for glioblastoma [52,53]. There are several cancer clinical trial studies utilizing TIM-3 inhibitors [54,55,56] and their success rates have been long suggested to be improved when used in combination with additional treatment such as PD-1/PD-L1 blocking agents [20,54,57,58,59]. Therefore, a multiple pathway approach may be the best way to utilize TIM-3 inhibitors for treating glioblastoma patients.

### 2.2. TIM-3 in Glioblastoma

Recently our group has critically reviewed the role of TIM-3 as a prognostic marker, its expression on immune cells, and the implication of anti-TIM-3 agents in glioblastoma [60]. We also discovered that innate and adaptive immune cells from glioblastoma patients are less functional in what could be a TIM-3-dependent manner. Stimulated NK cells and T cells from glioblastoma patients had higher percentage of TIM-3 positivity and reduced levels of activity markers (such as CD69 and IFN-γ) compared to those from age-matched healthy donor individuals [53]. Our findings on NK cells are supported by previous studies in glioma [61,62]. The increase in TIM-3 positivity on CD4+ and CD8+ T cells has been validated across many glioma studies including high-grade gliomas compared to low-grade gliomas or healthy donors [63,64]. Moreover, studies have reported an increase in TIM-3 on tumor T cells compared to peripheral T cells in matched glioma and glioblastoma samples [64,65,66]. Contrary to these findings, some studies have reported no difference in TIM-3 across glioblastoma T cells (tumor or peripheral) and healthy controls [62,67,68] which could be a result of tumor heterogeneity, differences in sample handling, and experimental design. Macrophages are another key immune subset that comprises a decent percentage of cells in the glioblastoma tumor microenvironment (TME). Several studies have shown that M2 macrophages, a suppressive pro-tumor cohort of macrophages, express higher levels of TIM-3 in glioma patients [61,69]. Mechanistic studies by Ni et al. and Zhang et al. have linked this M2 polarization and enhanced TIM-3 to PTEN (null) [25] and NFAT-1 (active) [70].

We have seen that patients also displayed a reduced expression of BAT3, resulting in upregulated TIM-3 expression and functionality on T cells [53]. Elevated levels of TIM-3 in glioblastoma patients were also found to positively correlate with tumor cell survival and tumorigenesis, as described by our team [53] and others [24,61,64,70,71,72,73,74]. When analyzed at a gene level, out of all the checkpoint molecules in the panel, including more studied markers such as *PD-1*, *PD-L1*, and *CTLA-4*, *HAVCR2* (TIM-3) expression was the highest in glioblastoma samples [24] and its high expression was a glioblastoma phenomenon and not seen for grade II and III tumors [74]. These findings may explain why anti-PD-1, anti-PD-L1, and anti-CTLA-4 treatments are not effective for glioblastoma patients [75,76,77,78,79,80], and why the potential use of anti-TIM-3 therapy may be a better approach to treat glioblastoma.

**Figure 1:**
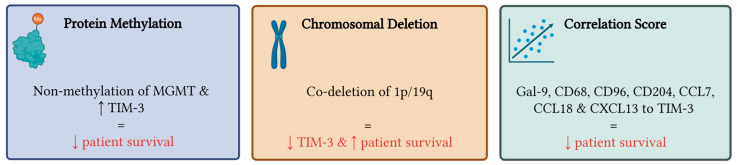
*The association of TIM-3 to glioblastoma processes.* TIM-3 protein is associated with several glioblastoma processes including protein methylation, chromosomal deletions, and expression and correlation scores, all of which are linked to patient survival. The non-methylation of O6 methylguanine-DNA methyltransferase (MGMT) and the increase in TIM-3 expression were linked to reduced patient survival, and the co-deletion of 1p/19q resulted in reduced TIM-3 expression and enhanced patient survival. The correlation scores between proteins such as Galectin-9 (Gal-9), CD68, CD96, CD204, CCl7, CCL18, and CXCL13 to TIM-3 were linked to reduced patient survival. Created in BioRender.

Further studies using elaborate data sets have described the potential association and therapeutic significance of TIM-3 with other glioblastoma processes (Figure 1). A link has been made between TIM-3 and protein methylation, chromosomal deletions, and expression and correlation scores, which are associated with glioma/glioblastoma survival. One study has shown that the non-methylation of a cytotoxicity inhibiting DNA repair protein—O6 methylguanine-DNA methyltransferase (MGMT) [81]—in combination with high TIM-3 expression correlated with reduced patient survival [74]. Moreover, the co-deletion of 1p/19q in gliomas resulted in the reduction of TIM-3 and galectin-9 expression which enhanced anti-tumor responses and thus improved patient survival [82]. Correlation studies have reported that TIM-3 expression levels in association with galectin-9 [25], CD68 monocyte/macrophages markers [69], CD96 checkpoint markers [83,84], CD204 tumor-associated markers [85], and CCL7, CCL18, and CXCL13 [86], result in poorer survival outcomes. These studies have linked TIM-3 to glioblastoma signatures and glioblastoma survival; however, whether these factors regulate each other and the mechanisms behind them remains unclear.

## 3. TGF-β

### 3.1. TGF-β Background

The transforming growth factor beta (TGF-β) family of cytokines are important cellular molecules responsible for a range of biological processes such as tissue homeostasis, repair, cellular support, and immunosurveillance [87]. TGF-β was initially discovered to be produced by transformed cells in 1978 [88]. During this era the different isoforms of TGF-β (TGF-β1, TGF-β2, and TGF-β3) were additionally discovered [89,90,91,92]. Many studies have shown that TGF-β can be secreted by a range of different non-transformed cells and tissues including the brain, heart, muscles, and lungs [93,94,95,96,97,98]. This ubiquitous expression of TGF-β highlights its key role in cellular processes. These processes include cell survival, metabolism, proliferation, growth, differentiation, adhesion, migration, apoptosis, and immune responses [99,100].

The TGF-β complex is formed by receptor–ligand binding, with three known receptors (TβRI, TβRII, and TβRII) binding to the three known TGF-β isoforms (TGF-β1, TGF-β2, and TGF-β3) in different combinations based on their affinity [90,101,102,103,104,105,106,107]. When inactive, TGF-β complex is stored in the extracellular matrix in a latent form [108]. When TGF-β is activated by factors such as integrins, acids and bases, ROS, TSP-1, and proteases, it removes itself from its latent complex and is released as a mature cytokine [109]. This process is necessary for signal transduction to occur and subsequent TGF-β signaling can be initiated in a canonical and non-canonical manner [109].

The canonical pathway is also known as the Smad-dependent pathway. Smads are transcriptional factors that are activated when heteromeric receptor complexes are formed on the cell surface [110]. TGF-β-TβRI-TβRII is an example of a heteromeric receptor complex and is formed by TGF-β binding to its receptor TβRII (x2), and the recruitment of TβRI (x2) [111]. This triggers a signaling cascade involving a series of phosphorylation steps, leading to the initiation of receptor-regulated Smads (R-Smads) (Smad2 and Smad3). R-Smads bind to Smad4, a co-Smad (common partner Smad), forming a complex that migrates into the nucleus [112]. In the nucleus, the Smad2/3/4 complex works in conjunction with a range of transcriptional factors and high-affinity DNA-binding coregulators to determine whether genes are switched on or off [113,114,115,116]. Inhibitory Smads (I-Smads) (Smad6 and Smad7) are another group of Smads that result in blocking R-Smad activation or the formation of the Smad2/3/4 complex. I-Smads are activated by TGF-β and bone morphogenic protein (BMP) signaling, working in a negative feedback loop resulting in Smad6 inhibiting BMP signaling and Smad7 inhibiting canonical and non-canonical TGF-β signaling [115,117,118,119].

Non-canonical Smad-independent TGF-β signaling can activate key cell signaling pathways such as ERK/p38 MAPK, JNK, GTPase, NF-κB, PI3K/AKT, and JAK/STAT [109,120,121]. These pathways are linked to cell proliferation, survival, migration, death, immunity, and so forth in diseases including cancer [122,123,124,125,126]. Zhang 2017 has published a detailed review on the non-canonical TGF-β signaling pathway [127].

The TGF-β cytokine is heavily altered by variation in physiological conditions and has very little ‘allegiance’ to its host [128]; therefore TGF-β can act as both a tumor suppressor and tumor promoter depending on the context [129,130]. This phenomenon is known as the ‘TGF-β paradox’. TGF-β is able to effectively inhibit uncontrolled growth and proliferation by suppressing the expression of c-Myc and cyclin dependent kinases (CDKs) and enhancing CDK inhibitors, stopping the progression of the cell cycle in these tumors [131]. Cell cycle arrest can be induced by TGF-β at any stage along with the increased transcription of cell cycle regulators, inhibiting the progression and function of various immune cell types [131].

Furthermore, TGF-β is a major contributor toward the progression of aggressive cancers and can instead act as a proto-oncogene when influenced by the TME [128,132]. The increased presence of TGF-β in tumors has been linked to increased levels of cellular transformation, angiogenesis and tumor growth, immune evasion, and metastasis via epithelial-to-mesenchymal transition (EMT) and endothelial-to-mesenchymal transition processes [133,134,135,136,137,138]. Tumor cells are known to evade the inhibitory effects of TGF-β through either mutation or the inactivation of TGF-β receptors by altering Smad pathways [139]. The recruitment of inflammatory cells and fibroblasts through TGF-β interacting with the TME may heavily contribute to the cancer’s aggressive phenotype [128].

Transforming growth factor-β is highly immunosuppressive with studies observing the inhibition of antigen presentation and the function of key innate NK cells [140,141]/DCs/neutrophils/macrophages [142,143,144,145,146] and adaptive (B cells/T cells [147,148,149]) immune cells. Its immunosuppressive nature is further highlighted by enhancing the expression of the suppressive transcriptional factor FOXP3 on Tregs [150,151], but also on CD8+ T cells [152], and skewing B cells in becoming suppressive Bregs [153,154].

Although studies have investigated the presence or absence of TGF-β signaling on tumorigenesis, tumor growth and tumor invasion, and the metastasis and composure of the TME, one key difficulty with using TGF-β therapies is the importance of TGF-β in a broad range of regular cellular processes such as embryonic development, wound healing, and tissue homeostasis, as reviewed by Deng et al. [109]. Despite there being potential for the use of these drugs, efficacy and safety issues are still of concern. Connecting multiple different cancer-related pathways for a multiangled treatment approach may be the best way of using these drugs.

### 3.2. TGF-β Signaling in Glioblastoma

Broader studies on the relationship between gliomas and TGF-β demonstrates similarities with other cancers, stating that TGF-β expression is increased in most gliomas and is potentially involved in tumor pathogenesis through the inhibition of anti-tumor immunity, and is thus linked to poor prognosis [155,156]. In glioblastoma, all three isoforms of TGF-β are expressed in the tumor and linked to poor prognosis. TGF-β1 and TGF-β2 are more highly expressed than TGF-β3, as TGF-β1 works in an autocrine loop to further increase TGF-β2, via CREB1 [157].

**Figure 2:**
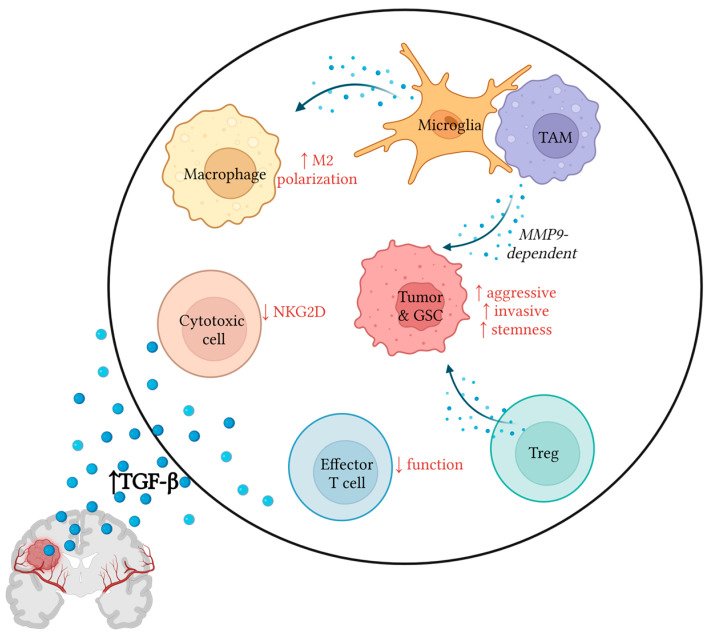
*The impact of TGF in the glioblastoma TME.* The key TGF-β-producing cell subsets in the TME include the tumor cells and GSCs, microglia, TAMs, and Tregs. TGF-β can inhibit the function of cytotoxic cells (downregulating NKG2D) and effector T cells (reducing anti-tumor immunity), and can increase tumor cell and GSC aggressiveness, invasiveness, and stemness. It additionally can increase the polarization of macrophages to an M2 phenotype, all of which contribute to the immunosuppression observed in the TME. Created in BioRender.

In terms of immunosuppression in the glioblastoma TME, many immune cell subsets are affected by TGF-β signaling. Figure 2 outlines the cells in the TME which produce TGF-β. Microglia produce TGF-β to promote the aggressive and invasive characteristics of glioblastoma cells [158,159]. Studies have shown that this can occur in a *MMP9*-dependent manner for glioblastoma stem cells (GSCs) when microglia and tumor-associated macrophages (TAMs) express TGF-β [160]. Furthermore, TGF-β1 is linked to M2 polarization and acts in a positive feedback loop through M2 macrophages releasing further TGF-β and enhancing stemness via EMT mechanisms [161,162]. The recruitment of Tregs is also enhanced in gliomas, with IL-17+ Tregs inhibiting CD8+ T cells, and the production of TGF-β by Tregs enriching GSCs and causing the glioblastoma cells to secrete IL-6 and enhance their stemness [163,164,165]. Moreover, TGF-β can downregulate key activating receptors on immune cells such as *NKG2D* expression on cytotoxic cells (NK cells and CD8+ T cells) and inhibit this process by reversing it [166,167]. These points highlight the ability of TGF-β to influence and regulate the immune cells in the glioblastoma TME.

In 2012, Kaminska et al. demonstrated the promise of TGF-β inhibitors as novel and potential anti-tumor therapies in addition to the standard care of TMZ down-regulating the expression of TGF-β at a protein level [156]. This study, however, was more applicable to broader gliomas and not directly to glioblastoma, though similarities in physiology remain relevant. Since then, there have been additional studies, albeit limited, investigating the manipulation of the TGF-β pathway for the treatment of recurrent glioblastoma. Conjugate in vivo TGF-β inhibiting mouse studies using TMZ with other agents such as perillyl (NEO212) halted the endothelial-to-mesenchymal process which contributed to tumor migration and evasion [168], and cholesterol lowering statins such as simvastatin reduced tumor margin invasion and prolonged survival [169]. Another benefit of targeting TGF-β signaling in glioblastoma is the inhibition of M2 macrophage polarization. Microglia and macrophages make up a large component of glioblastoma tumors and there is a known crosstalk between these cell populations [170]. Microglial-secreted TGF-β can cause the macrophages to polarize into M2s and thus work in a positive feedback loop to produce greater levels of TGF-β [170]. This is one key explanation as to how glioblastoma tumors are highly aggressive, through enhanced angiogenesis and invasion [171]. Immunotherapies have shown promise in solid tumors such as melanoma, head and neck squamous cell carcinoma, and non-small-cell lung cancer [172]; however, in glioblastoma it remains unsuccessful, a result of factors such as the BBB and the complexed TME. In 2021 a group investigated the potential use of inhibitors to αv integrin/TGF-β in aggressive GSCs, in combination with NK cell immunotherapy. NK cells within the tumor can interact with αv integrin in GSCs through CD9 and CD103 molecules, facilitating TGF-β1 secretion by GSCs thus inhibiting NK cell cytotoxicity [173]. Further to this, by replacing the glioblastoma NK cells with healthy NK cells with normal cytotoxic function in combination with galunisertib, a small molecule TGF-β receptor 1 inhibitor, mice survived for longer [173]. Results were even greater when TGFBR2-KO NK cells were used in this model [173]. In relation to this, a phase I clinical trial study currently in recruitment inserts engineered NK cells with deletions of TGF-betaR2 and NR3C1 (CB-NK-TGF-betaR2-/NR3C1-) into recurrent grade 4 astrocytoma (glioblastoma) patients to investigate its ability to control disease via anti-tumor functions (NCT04991870). Another phase I clinical trial study incorporating immunotherapy-based TGF-β treatment used M7824, a TGF-β and PD-L1 targeting fusion protein, for recurrent glioblastoma patients. This study observed that 8 out of 35 patients had successful disease control with feasible safety [174]. This shows that dual therapies incorporating checkpoint inhibitors with TGF-β therapy can be effective, however they need refinement to enhance success rates.

Other trial studies include a phase IIb using a TGF-β2 inhibitor AP12009 in combination with TMZ or PVC for recurrent high-grade gliomas which have been completed (NCT00431561). Moreover, the phase III study aimed to determine the safety and efficacy of AP12009 compared to standard chemotherapy in patients with recurrent or refractory anaplastic astrocytoma or secondary glioblastoma (NCT00716280); however it was terminated and therefore inconclusive. Two additional studies have used galunisertib in the context of recurrent glioblastoma and the other for newly diagnosed malignant glioma. NCT01582269 is an active study incorporating galunisertib with lomustine chemotherapy for the treatment of recurrent glioblastoma patients, and as it is an ongoing study the results are yet to be published. NCT01220271 combined galunisertib with radiation and TMZ for newly diagnosed malignant glioma patients and showed that despite the rate of disease control being better for patients treated with galunisertib plus radiation and TMZ compared to radiation and TMZ only, it did not change the survival outcome [175]. Similar to this trend, fresolimumab, an inhibitor for all three isoforms of TGF-β used in patients with glioma and glioblastoma, did not show favorable patient outcomes despite the drug being able to penetrate the high-grade gliomas (NCT01472731) [176].

A common theme across the studies manipulating the TGF-β pathway for treatment is that they are largely conducted in mouse models, and human studies have not progressed to advanced clinical trial stages. This emphasizes the need for more studies on the biology of human gliomas, specifically glioblastoma, for better treatment outcomes.

## 4. STING

### 4.1. STING Background

Discovered in 2008, stimulator of interferon genes (STING) and its pathway is a key innate and adaptive immunity activator and, as the name suggests, is important in the production of type I interferons and pro-inflammatory cytokines in different contexts [177,178,179,180,181,182]. The STING pathway is initiated via the detection of viral DNA, damaged self-DNA, micronuclei, and mitochondrial DNA (mtDNA) by a protein called GMP-AMP synthase (cGAS) [183,184,185]. A complex is formed with double stranded DNA (dsDNA) or mtDNA and cGAS, and this interaction results in a series of processes leading to the creation of cGAMP [178]. cGAMP is a secondary messenger which initiates STING protein to undergo a confirmational change and thus translocate from the endoplasmic reticulum (ER) to the Golgi [186,187], resulting in the recruitment of TANK-binding kinase 1 (TBK1) and IκB kinase (IKK). These kinases initiate the phosphorylation of IFN regulatory factor 3 (IRF3) and NF-κB inhibitor IκBα, respectively [188,189,190,191]. The phosphorylation of IRF3 causes the translocation of the protein into the nucleus where it activates type I interferon-related genes. The phosphorylation of IκBα fast-tracks the translocation of the NF-κB-initiated transcription of inflammatory cytokines such as IFNs, TNF, IL-1, and IL-6, and therefore inflammatory responses [182,192,193].

Stimulator of interferon genes can be expressed by different cell types including innate and adaptive immune cells [177,194,195,196], endothelial cells [197], epithelial cells [198], and neurons [199]. This means that the cell type and the disease it is activated in dictate what type of response it generates. STING plays a role in different health settings, including disease progression in chronic renal disease, acute kidney injury [200], and cardiovascular disease [201,202], and in neurological disorders such as neuroinflammation, pain, and brain injury [203]. In the context of the cancer TME, STING activation in immune and cancer cells has several anti-tumor responses. These consist of the polarization of TAMs to M1 macrophage phenotypes [204,205], inhibiting the activity of Tregs [180,205,206], and inhibiting myeloid-derived suppressor cells (MDSCs) [207,208,209], decreasing immunosuppression in the TME. Moreover, STING plays an important role in DC maturation [210,211,212], and the recruitment (via enhanced CXCL9, CXCL10, and CCL5) [213,214,215], activation, and function of T cells [216,217] and NK cells [214,218,219]. In tumor cells, STING activation regulates cell death via DNA damage responses, activating cell death pathways including CHK2-p53-p21 and ferroptosis [220,221,222], and via the recruitment and cytotoxic function of immune cells such as NK cells [223,224]. In certain circumstances STING signaling is linked to poor outcomes. In skin cancer STING aids in tumorgenicity [225] which can be attributed to chronic inflammation caused by the phagocytoses of dying cells, causing oncogenesis [226]. This overactive inflammatory response can cause increased Treg [227] and MDSC [228] infiltration, and highly induced immunoregulatory indoleamine 2,3-dioxygenase (IDO) enzymes [229]; these responses can lead to autoimmunity [230,231]. Moreover, high chromosomal instability and ongoing STING activation in tumor cells have been linked to tumor invasion and metastasis, a phenomenon linked to the switch of type I IFN and canonical NF-κB signaling to non-canonical NF-κB signaling [232]. This highlights the balance that is required for STING signaling to maintain an anti-tumor response.

The therapeutic role of STING agonists and cGAMP analogs, such as ADU-S100 and MK-1454 given as a single agent, have shown modest clinical benefits in patients with advances staged solid tumors or lymphomas [233,234,235]. This response was enhanced when given in combination with anti-PD-1 drugs [236], suggesting that STING agonists work best with checkpoint inhibitors, which could also translate to other checkpoint proteins such as TIM-3. STING agonists do come with some complications such as T cell death. High and/or repetitive doses of STING agonist can not only kill tumor cells, but also kill T cells and impair T cell tumor immunity [237]; hence, further study into the use of STING agonists, particularly in combination with additional drugs, is necessary.

### 4.2. STING Signaling in Glioblastoma

As STING can initiate immune responses its suppression in tumor cells is unsurprising due to the immune-evading nature of tumors [238,239]. This suppression is also apparent in glioblastoma, with epigenetic changes being reported, resulting in STING silencing; this silencing has been proposed as a feature of the glioblastoma cell of origin [240]. As previously mentioned, immunotherapy has shown promise in solid cancers such as melanoma [241]; however it remains unsuccessful in glioblastoma [78,242]. This is in part due to the complex TME and glioblastoma/gliomas being ‘cold’ immunological tumors [243,244]. STING expression is epigenetically silenced in glioblastoma tumor cells but can remain active in immune cells [240]; hence, promoting the STING pathway in tumors could be one way to address this issue as it could lead to enhanced immune stimulation.

A key study by Low et al. showed that the epigenetic silencing of STING via cg16983159 methylation in the brain resulted in the inactivation of the pathway [240] despite glioblastoma being rich in cytoplasmic extrachromosomal DNA [245]. The introduction of demethylating agents which inhibit DNA methyltransferase in vitro reversed this phenomenon and restored STING expression in glioblastoma cell lines [240]. However, this was not a tumorigenesis phenomenon as this hypermethylation of the STING promoter was also seen in normal brains and could be linked to brain development [240]. Despite this, across several human glioblastoma cell lines, CXCL10 expression was reduced [179]. CXCL10 is an immune cell attractant and expressed downstream of STING signaling, post IFN production, indicative of active signaling [246,247]. The activation of STING in gliomas can enhance anti-tumor immunity [248], and therefore several glioma/glioblastoma pre-clinical studies have explored ways to directly and indirectly activate the STING pathway via mechanisms including STING agonists, radiation therapy, electric field therapy, and oncolytic viruses [179,248,249,250,251,252]. Najem et al. have shown that STING agonists in combination with radiotherapy can reprogram the TME [250].

Reactivating the STING pathway has been proposed to sensitize glioblastoma tumors to immunotherapies [253]. The activation of STING pathways can be induced by the current treatment options for glioblastoma and can also enhance the efficacy of the drugs. An in vitro study has shown that TMZ in combination with an antibody against CD47, a molecule that is involved in inhibiting phagocytosis, resulted in ER-stress-activated STING. This caused a greater antigen presenting cell phagocytosis of tumor cells, and antigen cross-presentation and T cell priming, mechanisms resulting in enhanced immunity [254] which were only seen in the combination therapy group and not for anti-CD47 alone [254]. Furthermore, in glioblastoma cells that harbor PTEN mutation, STING agonists enhanced the efficacy of TMZ, leading to favorable survival [255].

**Figure 3:**
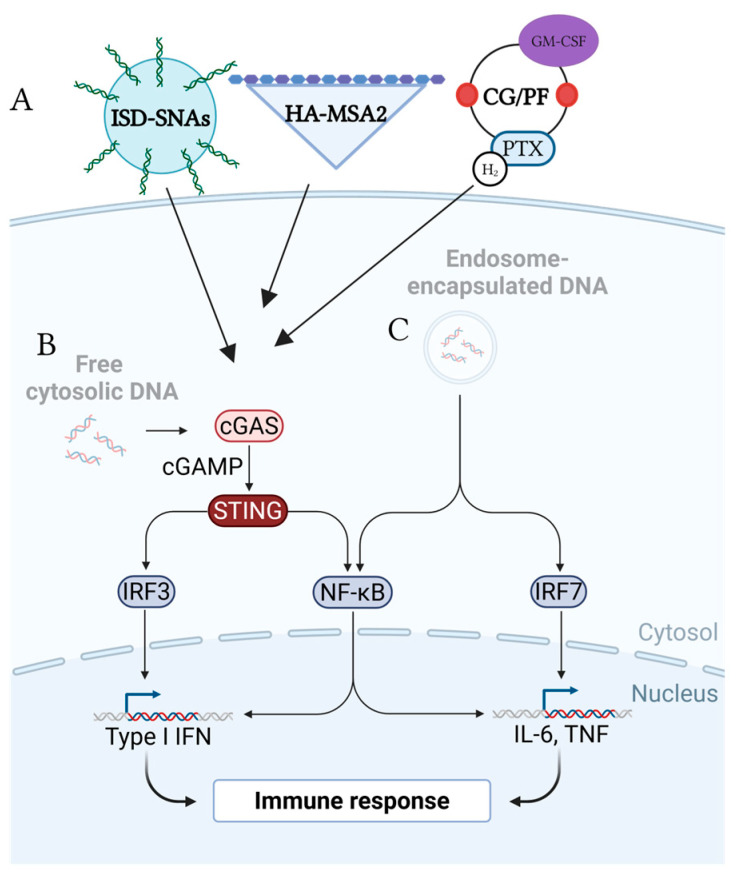
*Drug delivery systems using STING agonist for glioblastoma treatment.* Spherical nucleic acid-based immunotherapy called ISD-SNA, hyaluronic acid-based conjugate models with MSA2 STING agonist, and synthetic cyclic dinucleotide called CDA with GM-CSF using a paclitaxel (PTX) prodrug hydrogelator (CG/PF) (**A**) have been recently studied for their potentially enhanced ability to activate STING and downstream pathways for effective anti-tumor immune responses. These are three proposed delivery systems as well as (**B**). Free cytosolic DNA can activate cGAS/cGAMP, STING, and downstream proteins IRF3 and NF-κB, causing translocation into the nucleus, and the transcription of type I IFNs and cytokines such as IL-6 and TNF to initiate immune responses (**C**). Endosome-encapsulated DNA can directly activate NF-κB and IRF7 which in turn transcribe IL-6 and TNF in the nucleus and activate immune responses. Created in BioRender.

Drug delivery is an important avenue for therapeutic benefit in glioblastoma and has been investigated in the context of STING pathways across several studies. Mahajan et al. developed a novel spherical nucleic acid (SNA)-based immunotherapy called ISD-SNAs, which binds to cGAS and activates cGAS-STING-IRF3 pathways [256] (Figure 3). When administered into immunocompetent mice bearing glioblastoma tumors, ISD-SNA enhanced median survival compared to the control, and the combination of ISD-SNA with immune checkpoint inhibitors resulted in complete tumor regression [256]. Immunogenicity was confirmed via an increase in effector T cells, M1 macrophages, and activated NK cells and B cells, compared to mice given PBS or ssDNA-SNA, making these changes specific to ISD-SNA [256].

Another study has used a hyaluronic acid (HA)-based conjugate to locally deliver the STING agonist MSA2 with the aim of simultaneously activating innate and adaptive immunity in glioblastoma [257] (Figure 3). In vitro studies in tumor and immune cells in glioblastoma have shown that HA-MSA2 activates the STING and downstream genes after a short incubation period. Furthermore, the conjugation of HA to the MSA2 increased key cytokines and the expression of chemokines IFN-β, TNF-α, IL-6, and CXCL10 across DCs, macrophages, and microglia cells. In glioblastoma tumor cells HA-MSA2 can trigger cell death through the activation of STING, and in SB28 cells HA-MSA2 can cause ICD and thus release tumor-specific antigens which can be detected and targeted for treatment [257]. In vitro studies using HA-MSA2 in a mouse orthotopic model harboring SB28 tumors have shown reduced tumor growth and improved survival, features attributed to activated anti-tumor immunity [257].

Tumor-associated macrophages comprise ~30–50% of cells in gliomas [258] and TAMs are well known for their immunosuppressive nature in cancer including glioblastoma [258,259]. Li et al. have recently shown that TAMs in the TME transport lactate to GSCs through proton-coupled transporters MCT4-MCT1 [260]. Lactate is a glycolytic metabolite that can undergo epigenetic modifications such as lactylation and contribute to an immunosuppressive TME [261]. Single-cell RNA sequencing datasets of glioblastoma tumors were screened and analyzed for the enrichment of glycolysis pathways, and it was determined that TAMs were the cell subsets in glioblastoma that fit this profile. TAMs and tumor cells harbored a lactate transmembrane signature, suggesting a potential link between these cell populations in this specific pathway. This was further confirmed via the upregulation of lactate transmembrane protein-related genes SLC16A1 and SLC16A3 in glioblastoma compared to normal brain tissue when TCGA datasets were screened [260]. The lactate in GSCs causes them to become more proliferative and thus leads to cancer progression. It additionally induces the lactylation of the non-homologous end-joining protein KU70 at lysin K317, a process which inhibits cGAS-STING signaling and promotes immunosuppression in the TME [260]. Using an immunocompetent orthotopic xenograft model, the inhibition of lactate transportation or targeting the lactylation of KU70 in combination with anti-PD-1 checkpoint inhibition led to enhanced survival and reduced tumor mass, as compared to monotherapy and vehicle control. This combination therapy additionally increased CD8+ T cell infiltration and cytotoxic function and reduced the number of TAMs, transforming the TME to an anti-tumor environment [260].

A recent comprehensive study investigating localized drug delivery using STING agonists has demonstrated a potentially successful way to treat glioblastoma recurrence [262] (Figure 3). The study used a synthetic cyclic dinucleotide (CDN) called CDA, a STING agonist which can generate efficient immune activation in glioblastoma [262]. The localized co-delivery of CDA and GM-CSF using a paclitaxel (PTX) prodrug hydrogelator delivery system (CG/PF) achieved the activation of both innate and adaptive immunity [262]. The use of PTX in this model is linked to its known immunogenic cell death (ICD)-inducing properties which break down tumor cells and release tumor antigens. This promotes immunogenicity in cancer through enhanced DC antigen presentation and antigen-specific T cell activity, also seen in glioblastoma [263]. As high doses (100 nmol) of STING agonist have shown minimal efficacy in preclinical models [264], a low dose (14.5 nmol) approach was used for this study [262]. Key immune populations such as CD45+ cells, CD80+ DCs, and CD86+ DCs were more highly attracted to the tumor site in the CG/PF group compared to the saline control group, and the DCs were highly functional via their ability to process tumor antigens and the tumor specificity of CD8+ T cells into the tumor tissue. Furthermore, M1/M2 ratios were favorable and Treg population was reduced in the CG/PF group compared to the control. Tumor progression was assessed in a GL261 glioblastoma mouse model, with CG/PF-treated mice showing significantly reduced tumor progression and burden compared to PF monotherapy controls, and surgical resection in combination with treatment resulted in the tumor-free survival of these mice for up to 120 days [262]. The low doses of STING agonist used meant that this delivery system accomplished greater tumor regression and reduced rates of relapse [262]. The immunosuppressive TME of glioblastoma is one of the key challenges in cancer; thus reconfiguring the TME of glioblastoma to an immune active TME means that immune checkpoint inhibitors could also be more effective in glioblastoma. Shang et al. [262] along with other studies mentioned above, have shown this, through the therapeutic potential of CG/PF being further enhanced with the addition of checkpoint inhibitors via enhanced tumor control and immune cell memory. The results from this study highlight the potential of this specific delivery system in reconfiguring the glioblastoma immunosuppressive TME and thus further study in human trials are necessary to determine if these key findings are translatable.

Studies have shown promise with STING-inducing tumor responses; however a large percentage of STING agonist experiments in glioblastoma have been conducted on animal models, including mice and canines [248,265,266,267,268]. Deeper human studies are necessary to elucidate how STING can be incorporated for glioblastoma tumor immunity.

## 5. Interconnecting Pathways in Cancer

There are limited studies investigating the role of STING, TIM-3, and TGF-β pathways in not only glioblastoma, but cancer in general. Despite this, the overlap and interconnection between these pathways highlights the need for experimental exploration in this avenue for potential therapeutic outcomes.

### 5.1. STING and TIM-3

As mentioned above, recent studies have found that promoting STING pathway allows for enhanced checkpoint inhibitor efficacy in glioblastoma [256,260,262]. Studies have investigated the effects of STING agonists in combination with anti-TIM-3 therapy on tumors and have found an enhanced anti-tumor response in a DC and T cell manner. STING agonist ADU-S100 can cause an increase in TIM-3 expression on type 2 conventional dendritic cells (cDC2), which are a subset of immune cells that contribute to the immunosuppressive TME by inhibiting the function of CD4+ T cells in both humans and mice [213,269]. Inhibiting TIM-3 can activate cGAS-STING signaling and thus promote extracellular DNA uptake through the intratumoral cDC1s [213]. Luo et al. have shown that the combination therapy of anti-TIM-3 with ADU-S100 (STING agonist) resulted in cDC2 regulation and their ability to act in antigen presentation. In this context, this process caused the release of CD4+ T cells and enhanced their anti-tumor response, prolonging survival. This can be used as a positive indicator for immunotherapy responsiveness [269].

In a lung cancer model, berberine derivative (C51), an isoquinoline alkaloid with strong anti-tumor properties, was studied for its ability to regulate the cGAS-STING-TIM-3 molecular axis, for favorable patient outcomes [270]. This study showed that at low concentrations, C51 was able to inhibit cancer cell proliferation, and its anti-tumor effects came from the activation of cGAS-STING and the inhibition of TIM-3. Mechanistic studies revealed that the STING-TIM-3 axis was regulated by cGAS and that STING forms a bond with TIM-3 causing the molecule to degrade in a K48-dependant ubiquitination manner [270]. It is via this system that a reduction in the malignancy of lung cancer cells occurred, and this was further confirmed using subcutaneous xenograft mouse tumors [270].

Epigenetic changes are a hallmark of cancer [271,272,273] and can regulate both STING [240] and immune checkpoint inhibitors including TIM-3 [274,275]. Incorporating epigenetic modulating drugs could transform the TME and rewire these changes that occur to allow for reduced immunosuppression and enhanced anti-tumor responses, as reviewed by Dai et al. 2021 [276].

These studies highlight the relationship between STING and TIM-3 pathways and how there is a link between the two. Mechanistic studies are necessary to determine how these molecules and their pathways are involved with one another and what causes them to respond in the way that they do in cancers, including glioblastoma.

### 5.2. TGF-β and TIM-3

Both TGF-β and TIM-3 are two key immune evasion tumor drivers. An early study observed the increase in TIM-3 expression on circulating monocytes and TAMs post TGF-β treatment in hepatocellular carcinoma (HCC) [277]. Additionally, the in vitro blocking of IL-6 in HCC cells reversed the effects of TIM-3 on cellular growth [277]. TGF-β can induce IL-6 in many cell types such as human lung fibroblasts [278]. Moreover, this study found a Smad-binding site in the 5′ region of TIM-3 which may suggest a direct signaling link with TGF-β, and a luciferase assay confirmed TIM-3 promotor activity by TGF-β [277].

In colorectal cancer Katagata et al. have reported a robust correlation between TGF-β-dependent active TMEs and *HAVCR2* expression [32]. This correlation was made by analyzing ~2200 colorectal tissues, organoids, and xenografts using genetic and protein-based methods. Diving deeper, in tumors, infiltrating M2-like macrophages expressing TIM-3 correlated with high TGF-β-activated stroma regions, with the stroma confirmed via VCAN positive staining. Further correlations were made between the expression of *TGFB* and *HAVCR2* genes and M2 macrophages, and the upregulation of TIM-3 on M2-polarized macrophages and TGF-β-stimulated monocytes were reported. These studies have shown a connection between multiple tumor promoting pathways, and that a TGF-β rich milieu can in part induce TIM-3 expression on immunosuppressive cell subsets such as M2 macrophages and TAMs [32], as confirmed in other cancer models such as liver cancer [279]. Wiener et al. have additionally demonstrated this trend seen in human mast cells where TIM-3 expression was upregulated by TGF-β1 [280]. TGF-β regulating TIM-3 could be a myeloid cell phenomenon. Further studies are necessary to conclusively confirm whether this is the case.

This phenomenon can be present in glioblastoma as TGF-β levels are high, M2 macrophages comprise a decent percentage of the cellular population, and TIM-3 signaling is dysregulated [53,60].

### 5.3. STING and TGF-β

**Figure 4:**
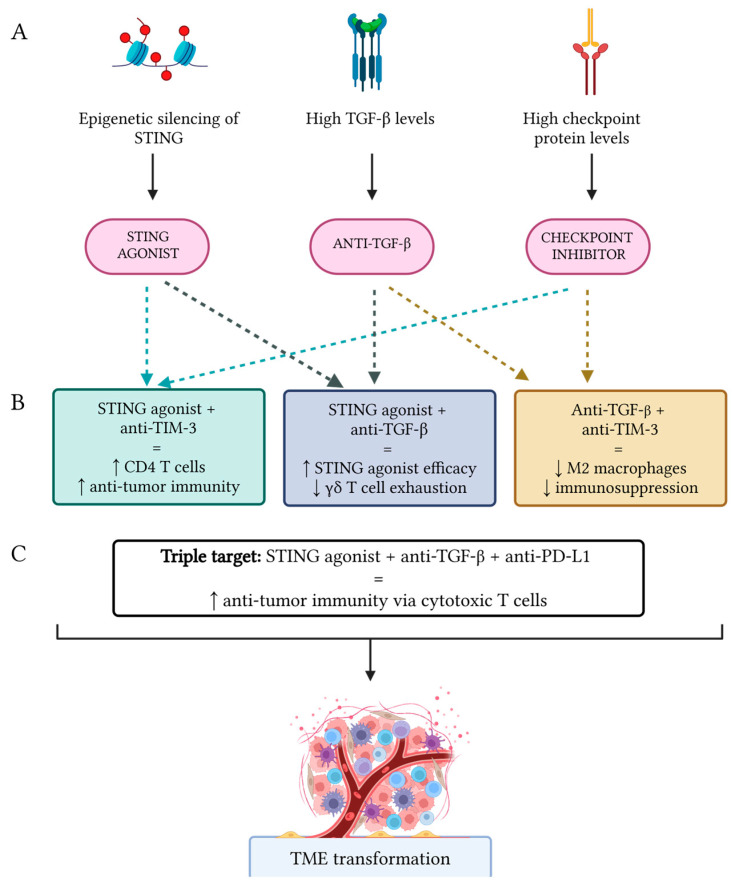
*Potential outcomes for the combinational use of STING*, *TGF-β*, *and TIM-3 drugs in glioblastoma.* (**A**) The epigenetic silencing of STING, elevated levels of TGF-β, and checkpoint proteins such as TIM-3 are key features of cancer including glioblastoma. (**B**) The combination of STING agonists with anti-TIM-3 can enhance anti-tumor immunity via increasing CD4+ T cells, STING agonists with anti-TGF-β can enhance the efficacy of STING agonists and reduce exhaustion of anti-tumor T cells (γδ T cells), and the combination of anti-TGF-β and anti-TIM-3 reduces the polarization of M2 macrophages and reduces overall immunosuppression. (**C**) The triple therapy of STING agonists, anti-TGF-β, and anti-PD-L1 (antibody–drug conjugate) has shown promise in pre-clinical cancer models and could potentially be the approach for glioblastoma to enhance anti-tumor immunity in a cytotoxic T cell manner. Utilizing drugs (STING agonists, anti-TGF-β, and checkpoint inhibitors) as dual and triple-pronged therapy has shown the potential to re-transform the immunosuppressive TME, which could potentially be the case for glioblastoma. Created in BioRender.

Luo et al. 2025 have extensively studied the interconnection and correlation between STING and TGF-β pathways, linking them to cancer progression. A key immunoregulatory subset of cells, Vδ2 γδ T cells, from lung cancer patients expressed lower levels of STING compared to healthy donors, and IFN-γ expression was diminished in these cancer patients, a feature that could not be reversed in the presence of STING agonist diABZI-C3 [281]. During early tumor development, tumor-derived cGAMP activated γδ T cells in a STING-IFN-γ-dependent manner [281]. TGF-β had minimal effects on cGAS and cGAMP levels; however it could downregulate STING expression [282], thus Luo et al. hypothesized that advanced stage tumor TGF-β is what causes the reduction in STING expression. Further to this, Luo et al. discovered that TGF-β is not only key in downregulating IFN-γ and STING, it also causes the exhaustion of γδ T cells, rendering them functionally inactive. When introducing TGF-β blocking agents, STING function and STING agonist efficacy were enhanced in advanced staged tumors in a γδ T cell manner. This is particularly interesting as it could be linked to TIM-3, and shows a potential connection between TGF-β, STING, and TIM-3 in cancer progression and poor patient survival (Figure 4). This connection between TGF-β, STING, and checkpoint inhibitors (PD-L1) has been published recently by Yi et al., showing that the triple targeting of these pathways, activating STING and inhibiting TGF-β and PD-L1 enhanced anti-tumor immunity via the recruitment of cytotoxic CXCR6+ T cells via the enhanced expression of CXCL16 in macrophages and DCs in an EMT-6 tumor bearing mouse model, as determined by scRNA-seq data [283]. This phenomenon could also be relevant to glioblastoma as TGF-β levels have been proven to be elevated in advanced disease [284] and STING expression is reduced [240].

Another connection between STING and TGF-β is through the protein STAT3. In small cell lung cancer, the STAT3-STING-IFN axis has been published as a controller of cancer spread [285]; thus STAT3 expression is necessary for STING signaling. Pei et al., however, have shown that in ThP1 cells, STAT3 inhibition via a small molecule inhibitor (HJC0152) in the presence of STING agonist diAM (PS)_2_ significantly elevated the expression of IFN-β in a dose-dependent manner [206]. It should be noted that the first study did not include a STING agonist and Pei et al. have shown that the HJC0152 activation of STING is not a direct response, rather it amplifies the effect of diAM (PS)_2_. Pei et al. have additionally suggested that the feedback activation of STAT3 via STING signaling could be causing immunosuppression, as STAT3 is a key molecule associated with tumorigenesis [206]. TGF-β is also linked to tumorigenesis and is not only an activator of STAT3 [286] but can also be activated by STAT3 as a STAT3 pathway is necessary for TGF-β-induced EMT [287,288]. This tumorigenic response of STAT3/TGF-β in glioblastoma is highlighted by the dual signaling promoting TMZ resistance through enhanced cellular senescence [289]. As STAT3 can be linked to STING and TGF-β, there could be a tumorigenesis connection in this axis, and this is worth exploring and understanding.

## 6. Limitations of TIM-3, TGF-β and STING Therapies

Despite the promise of targeting TIM-3, TGF-β and STING, there are some individual limitations associated with these therapies which need to be taken into consideration and addressed.

The efficacy of checkpoint inhibitors, including TIM-3, is dependent on several factors. Some factors which can contribute to the ineffectiveness of checkpoint inhibitors in cancer including TIM-3 are: administration as a monotherapy as compensation can occur when one checkpoint protein is silenced [290], and whether the tumor is labeled as ‘cold’ based on low immune infiltration [291]. In the triple regimen proposed in this review, the checkpoint inhibitors are introduced as the third target post anti-TGF-β and STING agonist, which could potentially overcome the regular limitations due to the initiation of an anti-tumor immune response.

Despite demonstrating promise in pre-clinical assessment, TGF-β inhibitors do not improve clinical outcomes. This can be attributed to several reasons, including the potentially opposing roles of TGF-β in different stages of cancer invasion/metastasis, dynamic TGF-β signaling, and protection from inhibitors when TGF-β signaling occurs through exosomal mechanisms, as is often the case in the cancer setting [292].

STING agonists may contribute to neurotoxicity, chronic pro-tumor inflammation, dosing towards pro-tumor outcomes, drug stability issues, and BBB limitations as reviewed by Wang et al. [293] and Ioannou et al. [294]. Some factors include the chronic activation of cGAS-STING in the normal cells of the central nervous system that may cause the overproduction of STING-associated Type I IFNs, TNF, CXCL10, and CCL5, resulting in neurotoxicity [295,296]. The activation of STING in the normal neural cells needs to be studied further and avoided when administering STING agonists. As previously mentioned, STING signaling can induce STAT3 expression caused by the chronic production of IL-6, a cytokine produced by STING signaling, also causing the upregulation of PD-L1 and contributing to an immunosuppressive TME [293]. Understanding the dosing window of STING agonist is important in potentially addressing this, as different phases of STING activation dictate whether an anti-tumor or pro-tumor response is achieved [293].

## 7. Conclusions

In conclusion, this review proposes the benefit of targeting the key signaling pathways TIM-3, TGF-β, and STING to reconfigure the glioblastoma TME, hence improving patient outcomes. There are several signaling pathways associated with glioblastoma progression, such as EGFR, NF-κB, Wnt, and PI3K/AKT/mTOR. TIM-3 and TGF-β are immune-evading pathways, and STING activates anti-tumor immunity. Immune evasion is an integral component of the suppressive glioblastoma TME, and the reactivation of key immune subsets along with the inhibition of suppressive cell activity is necessary to drive anti-tumor immunity.

Although some evidence is provided in the literature regarding cross-talk between these pathways in other cancers, very little literature focuses on the interaction of these pathways in the glioblastoma setting and thus we propose that more research into this work is required to further understand the complex signaling networks that promote glioblastoma progression. Moreover, disappointingly very little data is currently published which looks at the translational impact of these pathways in the glioblastoma setting nor the clinical evaluation of inhibitors to these pathways, highlighting the need for further work in this area to improve the overall understanding of glioblastoma progression and eventual inhibition.

Recent studies have shown that the drug delivery system used to regulate the signaling axis could be of benefit, providing greater drug success and additional anti-tumor responses. Initial in vitro studies incorporating TIM-3 and TGF-β inhibitors along with STING agonists in glioblastoma are necessary. Immune-profiling key immune subsets mentioned above are necessary to determine the effects of manipulating these signaling pathways in vitro on the immunity of the patient. Furthermore, it is important to investigate other aspects of the TME such as the tumor cells and supporting cells and their function to also address the potential limitations of the therapies mentioned above. These studies are needed to fully map the cancer associated pathways and their interconnected aspects for the improved treatment of glioblastoma. By dissecting the TME before and after this targeting regimen and determining whether the reconfiguration of the glioblastoma TME skewing towards an anti-tumor phenotype occurs, clinical trial studies can be designed and could possibly lead to an improved way to treat glioblastoma.

## Data Availability

No new data were created or analyzed in this study.

## Abbreviations

The following abbreviations are used in this manuscript:

AKT	Protein kinase B
BAT3	HLA-B associated transcript 3
BBB	Blood–brain barrier
BMP	Bone morphogenic protein
CDK	Cyclin dependent kinase
CDN	Cyclic dinucleotide
CEACAM-1	Carcinoembryonic antigen-related cell adhesion molecule-1
cGAS	GMP-AMP synthase
Co-Smad	Common partner Smad
DC	Dendritic cell
EGFR	Epidermal Growth Factor Receptor
EMT	Epithelial-to-mesenchymal transition
Gal-9	Galectin-9
GSC	Glioblastoma stem cell
Gy	Gray
HA	Hyaluronic acid
HMGB1	High mobility group box 1
ICD	Immunogenic cell death
IDH	Isocitrate dehydrogenase
IDO	Indoleamine 2,3-dioxygenase
IFN-γ	Interferon-gamma
IKK	IκB kinase
IRF3	IFN regulatory factor 3
I-Smad	Inhibitory Smad
LCK	Lymphocyte-specific protein tyrosine kinase
MAPK	Mitogen-activated protein kinase
MDSC	Myeloid-derived suppressor cell
MGMT	O6 methylguanine-DNA methyltransferase
mtDNA	Mitochondrial DNA
mTOR	Mammalian target of rapamycin
NF-κB	Nuclear factor kappa-light-chain enhancer of activated B cell
NK	Natural killer cell
OS	Overall survival
PI3K	Phosphoinositide 3-kinase
PtdSer	Phosphatidylserine
PTX	Paclitaxel
RB	Retinoblastoma
R-Smad	Receptor-regulated Smad
SNA	Spherical nucleic acid
STAT3	Signal transducer of transcription 3
STING	Stimulator of interferon genes
TAM	Tumor-associated macrophage
TBK1	TANK-binding kinase 1
TGF-β	Transforming growth factor-beta
TIM-3	T-cell immunoglobulin and mucin-domain containing-3
TME	Tumor microenvironment
TMZ	Temozolomide
Treg	Regulatory T cell
Tyr	Tyrosine
